# Development of a whey-based beverage with enhanced levels of conjugated linoleic acid (CLA) as facilitated by endogenous walnut lipase

**DOI:** 10.1016/j.fochx.2022.100547

**Published:** 2022-12-21

**Authors:** Mona Moslemi, Ali Moayedi, Morteza Khomeiri, Yahya Maghsoudlou

**Affiliations:** Department of Food Science and Technology, Gorgan University of Agricultural Sciences and Natural Resources, Gorgan, Iran

**Keywords:** Conjugated linoleic acid (CLA), Fermentation, Whey beverage, Lipolysis, Optimization, Taguchi

## Abstract

In this study, optimization of fermentation conditions, and applying endogenous walnut lipase were investigated for the manufacture of a fermented, whey-based beverage containing conjugated linoleic acid (CLA). Among different commercial starter and probiotic cultures, the culture containing *Lactobacillus delbrueckii* subsp. *bulgaricus* and *Streptococcus thermophilus* showed high potency for CLA synthesis. The fermentation time and the type of walnut oil (lipolyzed or non-lipolyzed) had significant effects on CLA production, as the highest CLA content (36 mg/g fat) was synthesized in the sample containing 1 % lipolyzed walnut oil fermented at 42 °C for 24 h. Moreover, fermentation time had the highest contribution on viable cell counts, proteolysis, DPPH scavenging activity, and final pH. A significant and positive correlation between cell counts and CLA content was also observed (r = +0.823, p < 0.05). This study establishes a cost effective approach for converting cheese whey to a value added beverage enriched with CLA.

## Highlights

• A whey-based beverage with enhanced levels of CLA was manufactured.
• Fermentation time was the most effective factor on CLA synthesis.
• Endogenous walnut lipase was applied as a cost-effective approach for increasing the CLA content.

## 1 Introduction

Conjugated linoleic acid (CLA) is a spatial and geometric isomer of linoleic acid (18: 2C, *cis*-9, *cis*-12) with many health-promoting effects such as anticancer, antioxidant, antidiabetic, and anti-obesity activity (Chin et al., 1992, Koba and Yanagita, 2014, Moya-Camarena et al., 1999). CLA is naturally found in meat and milk fat as a minor fatty acid. Nowadays, pure CLA is provided as dietary supplements which are mainly synthesized via alkaline isomerization of linoleic acid. However, there is concerns on chemical synthesis, since a mixture of different isomers is produced whose effects on human health are not completely known. Therefore, applying biological methods that lead to the production of predicted isomer(s) is of great importance (Farmani et al., 2010).

Some microorganisms have been identified with the ability to produce CLA, among them lactic acid bacteria (LAB) are preferred because they are generally recognized as safe (GRAS) (Liu et al., 2021). When applied in food fermentation, they can produce various beneficial metabolites such as CLA and other fatty acid metabolites (Aziz et al., 2022, Aziz et al., 2022). Regard this, during the recent years, LAB species have been used as starters or co-cultures to manufacture CLA-enriched yogurt, milk, meat, cheese, and soy milk (Das et al., 2005, Gorissen et al., 2012, Li et al., 2012). One of the main challenges in developing CLA-rich foods is that many organisms are not able to use esterified Linoleic acid (LA) as CLA precursor, and need free LA. In this term, the use of exogenous lipase in the process has been useful to liberate LA and therefore to enhance CLA synthesis in the final fermented product (Vahvaselka & Laakso, 2010). However, a more economic approach is to activate the endogenous lipase of the LA-rich substrate (Andrade et al., 2012).

Cheese whey, the major byproduct in dairy plants, is a desirable and cost-effective choice to be converted to value-added products such as fermented beverages (Karimian et al., 2020). Whey-based beverages and fermented/flavored dairy beverages containing cheese whey are popular with consumers around the world (Chavan et al., 2015). In addition, many researches have been done to naturally fortify milk-or whey-based products with bioactive ingredients (Khanlari et al., 2021, Özer and Kirmaci, 2010). According to our knowledge, no (or minor) studies have been performed to produce a CLA-rich fermented whey drink. Also, the use of endogenous lipase as a cost effective method for supplying CLA precursor has less been studied. The aim of this study was to optimize the fermentation conditions for the manufacture of a whey-based beverage rich in CLA using Tagouchi design. At first, some commercial starter and probiotic cultures available in the Iranian market were screened according to their ability for CLA synthesis, and the most potent culture was selected. For facilitating the CLA formation, endogenous walnut lipase was activated, and its effect on LA liberation was monitored. Then, the effects of different factors (the concentration and type of walnut oil used in the formulation, fermentation time, and incubation temperature) on the CLA concentration, final pH, proteolysis, and cell counts were evaluated. Finally, the optimum levels were determined. This study introduces the technical knowledge for the manufacture of a novel functional food from cheese whey.

## 2 Materials and methods

### 2.1 Chemicals and reagents

Pure linoleic acid was purchased from Acros Organics (NJ, USA), and l-serine from Biobasic Co. (Canada). DPPH and o-phthalaldehyde (OPA) were obtained from Sigma-Aldrich Co. (Steinheim, Germany). Dithioteritol (DTT), and other chemicals were purchased from Merck Co. (Darmstadt, Germany).

### 1.2 Microbial cultures

Commercial starter and probiotic cultures as single or combined cultures including Lyofast MOT 092 EE (containing *Lactobacillus helveticus, Lactococcus lactis* subsp*. lactis*, *Lactobacillus helveticus, Streptococcus thermophilus, Lactobacillus delbrueckii* subsp*. bulgaricus*), Lyofast BLC1 (*Bifidobacterium animalis* subsp*. lactis*), Lyofast LA3 (*Lactobacillus acidophilus*) and Lyofast Y259A (containing *Streptococcus thermophilus* and *Lactobacillus delbrueckii* subsp*. bulgaricus*) were kindly donated by Lactoprot Pishgaman (the sale representative of Sacco Company, Italy) as lyophilized powder. Also, pure cultures of *S. thermophilus* and *L. delbrueckii* subsp*. bulgaricus* were isolated from Lyofast Y 259 A. *L. rhamnosus* GG (ATCC 53103) and *L. paracasei* were obtained from Chr. Hansen's company (Denmark).

### 1.3 Activation of bacterial cultures

Direct Vat Set (DVS) cultures were added to sterilized milk, fast frozen by use of liquid nitrogen for the preparation of a stock culture, and then stored at −20 °C until use. The bacterial strains were activated by sequential sub-culturing of stock cultures in De Man, Rogosa and Sharpe (MRS) broth followed by incubation at 37 °C for 24 h. For *B. animalis* subsp. *lactis,* 250 µL of l-cysteine (1 %) was added to 5 mL of MRS medium. Pure single culture of *Streptococcus thermophilus* or *L. delbrueckii* subsp. *bulgaricus* was prepared by culturing Lyofast Y 259 on MRS agar or M17 agar, respectively. The pure cultures were validated morphologically.

### 2.4 Screening for CLA-Producing strains

The screening for CLA-Producing strains was performed similar to the method described by Ribeiro et al. (2018) with some modifications. Briefly, activated bacterial culture was centrifuged at 5000 rpm for 5 min, the supernatant was discarded, and the pellet was re-dissolved in sterile peptone water until a bacterial suspension with cell density around 3 × 10^8^ CFU/mL was obtained. Bacterial suspension was inoculated (1 %, v/v) into MRS broth containing different concentrations of LA (0.0, 0.25, and 0.5 mg/mL), and incubated for 48 h at 37 °C. After incubation, 1.5 mL of culture medium was centrifuged for 7 min at 13000 × g and then, the supernatant was mixed with 2 mL of isopropanol, and kept at ambient temperature for 3 min. Then, 1.5 mL of hexane was added to the solution, shaken well, and kept at ambient temperature for 5 min. The absorbance of upper layer was measured at 233 nm. The difference between the absorbance of solutions obtained from cultures with LA and that of control was determined, and regarded as CLA formation. Higher difference in absorbance showed higher concentration of CLA. The results were confirmed by Gas chromatography (GC). Finally, a commercial culture with the highest absorbance at 233 nm was selected for further experiments and production of fermented whey-based beverage.

### 2.5 Formulation of whey-based beverage

The beverage formulation contained low-fat UHT milk, skim milk powder and sweet cheese whey. A 16- run mixture design, provided by Design-expert software, was used for the optimization of beverage formulation (data not shown), in which the solid non-fat (SNF) was adjusted to 10 %. Formulation containing 50 mL of whey, 25.4 mL of UHT milk and 4.60 g of skim milk powder was considered as the optimum formulation in terms of texture, taste and the predominant proportion of whey. The selected formulation was added with appropriate amount of walnut oil, and used as the base for the manufacture of a fermented whey-based beverage enriched with CLA.

### 2.6 Production of lipolyzed walnut oil

In order to facilitate LA bioconversion to CLA, and to evaluate the effect of lipolysis on CLA synthesis, walnut oil was lipolyzed by endogenous walnut lipase similar to the method of Vahvaselka & Laakso (2010) with slight modifications. The milled walnut kernels was conditioned by adding deionized water to reach water activity (aw) of 0.70, and stored at ambient temperature for 12 days. Lipolysis progress was monitored by measuring the acid value of cold-extracted oil at different time intervals (0, 6 and 12 days). Also, microbial total count was determined in the milled walnut.

### 2.7 Manufacture of fermented whey-based beverage enriched with CLA

A mixture of cheese whey, low-fat milk, and skim milk powder (as mentioned above), selected as the product base, was added with commercial emulsifier (0.22 %, w/v), and then sterilized at 110° C for 15 min. Then, walnut oil (0.5 or 1 %, v/v) was added, and the mixture was homogenized at 10000 rpm for 3 min (Heidolph, Germany). After that, an appropriate amount (according to the manufacturer instruction) of selected starter culture was inoculated into the mixture, and incubated at 42 or 37 °C. Sampling was performed at time intervals according to Taguchi design.

### 2.8 Optimization of CLA synthesis using Taguchi method

In this study, Taguchi method was used to optimize the synthesis of CLA in a whey-based fermented beverage. As shown in Table 1, oil concentration, fermentation time, oil type, and fermentation temperature were considered as variables. Eight experimental runs (L8 orthogonal array) were designed by Taguchi method (Table 1).

**Table 1:** Results of Orthogonal Array L8 (41 X 23) designed by Taguchi.

Treatment	Fermentation time (h)	Oil type	Oil concentration (%)	Fermentation temperature	CLA	pH	Cell Counts	Antioxidant activity (%)	Proteolysis (l-serine, mg/ml)
				(°C)	(mg/g fat)				
1	6	Lipolyzed	0.5	37	21	5.8	6.64	25.2	0.13
2	6	Non-lipolyzed	1	42	12	5.79	6.54	27.5	0.22
3	12	Lipolyzed	0.5	42	21	4.79	7.11	27.1	0.49
4	12	Non-lipolyzed	1	37	17	4.66	7.09	28.9	0.4
5	18	Lipolyzed	1	37	30	4.61	7.48	33.2	0.49
6	18	Non-lipolyzed	0.5	42	27	4.72	7.34	29.8	0.6
7	24	Lipolyzed	1	42	36	4.69	7.36	40.4	0.46
8	24	Non-lipolyzed	0.5	37	31	4.45	7.56	37.5	0.3

### 2.9 Final product analysis

#### 2.9.1 Changes in pH and proteolysis

The pH values of the samples were determined at time intervals according to Taguchi design. The extent of proteolysis was determined similar to the method previously described by Donkor et al., 2007, Osuntoki and Korie, 2010 using o-phthalaldehyde (OPA) reagent. In this method, the reaction of free amine groups with OPA results in increase in absorbance at 340 nm. The results were reported as mg equivalent of l-serine per mL. A standard curve from different concentrations of l-serine was prepared for quantification (0.25 – 4.0 mg/ml).

#### 2.9.2 Determination of CLA content

Lipid fraction containing CLA was extracted similar to the methodology described by Li et al. (2012). Fatty acid methyl esters (FAME) was prepared by adding 1 mL of 15 % boron trifluoride (BF_3_) in methanolic solution followed by incubating in a water bath (50 °C) for 30 min. After that, 0.2 mL of hexane and 0.1 mL of distilled water were added, and the resulted mixture were centrifuged at 2000*×g* for 3 min. FAMEs were analyzed by a GC apparatus equipped with a HP-88 column (100 m × 0.25 mm i.d., 0.2 μm), and flame ionization detector (FID). Helium was used as the carrier gas at flow rate of 1 mL/min. The initial temperature for the column and detector were 250 °C and 280 °C, respectively. The temperature program started at 120° C (for 15 min), then increased to 180 °C (4 °C/min), and finally increased to 230° C, in which held for 16 min. Quantitative and qualitative analysis of CLA was performed by considering the chromatogram obtained from injecting pure cis-9, *trans*-11 CLA. CLA content was expressed as mg/g fat.

#### 2.9.3 Evaluation of DPPH radical scavenging activity

DPPH radical scavenging activity of the samples was evaluated similar to the method described by Osuntoki & Korie (2010). Briefly, 50 μL of the sample was added to 2 mL of methanolic DPPH solution (24 mg/L), mixed well, and stored in a dark place at ambient temperature for 30 min. Then, it was centrifuged at 4000 rpm for 10 min and absorbance of the supernatant was measured at 517 nm. The control sample was prepared in the same manner, except that distilled water was used instead of fermented beverage. The DPPH radical scavenging activity was calculated as follows:

$$DPPH\;scavenging\;activity\;\left(\%\right)=\frac{A_{c}-A_{s}}{A_{c}}$$

where A_c_ and A_s_ are absorbance values of control and sample, respectively.

#### 2.9.4 Bacterial viable cell counts

The growth of starter cultures was evaluated via enumeration of viable cell counts (Gül et al., 2005). Serial dilutions were prepared by adding 1 mL of sample to 9 mL of peptone water. Then, 1 mL of two appropriate dilutions were poured plated in MRS agar, and incubated under aerobic conditions at 37 °C for 72 h. Then, the cell counts were determined by the following equation:

$$N=\frac{\Sigma Ci}{V(n_{1}+0.1n_{2})d}$$

where C is the sum of counted colonies, V is the inoculated volume, n1 and n2 are the number of plates counted at the first and second dilution, respectively and d is the lowest dilution.

### 2.10 Statistical analysis

Taguchi method was used for the experimental design, and statistical analysis was carried out by Qualitek 4 software. In the screening step and preparation of lipolyzed walnuts oil, the comparison of mean values was performed by Duncan’s test (at 95 % confidence level) using Statistical Analysis Software (SAS, v.9.3).

## 3 Results & discussion

### 3.1 Screening for CLA-producing strains

In the first step of this study, two different concentrations of LA (0.25 and 0.5 mg mL^−1^) were used to screen bacterial strains with CLA-producing activity (data shown as supplementary material). The difference between the absorbance of organic layer obtained from the fermented samples containing LA with that obtained from the fermented control sample (without LA) was measured at 233 nm, and considered as CLA producing activity; the higher the absorbance at 233 nm, the higher is the ability of cultures in CLA production. High concentrations of free LA may have toxic effects on bacteria, while lower concentration stimulates the expression of linoleate isomerase and CLA production (Andrade et al., 2012). Overall, it was found that the lower LA level was more effective in producing CLA. Among different starter and probiotic cultures tested, Lyofast Y 259A (containing *S. thermophilus* and *L. delbrueckii* subsp*. bulgaricus*) displayed the highest ability in the production of CLA. The CLA synthesis potential of the mentioned culture was confirmed by GC analysis. However, it could be worthwhile to note that the strains did not show considerable ability in the production of CLA when used as single cultures. CLA production depends on multiple factors including temperature, bacterial strains, type and concentration of LA source (Devi and Rashmi, 2017, Özer and Kılıç, 2021). In accordance to our results, Lin et al., 2005, Van Nieuwenhove et al., 2007 revealed the potential of *L. delbrueckii* ssp. *bulgaricus* and *S. thermophiles* in CLA production. The ability of other LAB strains such as *L. plantarum* in CLA synthesis has also been reported by Ares-yebra et al., 2019, Li et al., 2012, Ribeiro et al., 2018.

### 3.2 Preparation of lipolyzed walnut oil

Partially lipolyzed walnut oil by endogenous lipase was provided as the substrate for production of CLA by selected starter culture in the fermented whey-based beverage. The acid value of the extracted oil increased markedly from 0.38 to 1.56 % as the storage time increased to 14 days (p < 0.05) (supplementary material). On the other side, total counts showed no significant changes (3.85 to 3.94 CFU/g) during storage (p > 0.05). This could be mainly due to the fact that such a_w_ was suitable for lipase activity, but unsuitable for microflora growth. Similarly, Vahvaselkä et al. (2006) exploited endogenous oat lipase to increase free LA concentration for CLA synthesis using *Propionibacterium freudenreichii *ssp. *shermanii*. Moreover, in another study, Vahvaselka & Laakso (2010) produced CLA in a mixture of camelina meal and okara by an oat-assisted microbial process and found that the CLA content was dependent on the substrate type and hydrolysis time.

### 3.3 Characterization of fermented whey-based beverage according to Taguchi design

The results obtained from the experimental design by Taguchi method for 8 experimental treatments are provided in Table 1. The effects of fermentation time at 4 levels (6, 12, 18, and 24 h), oil type at two levels (lipolyzed and non-lipolyzed), oil concentration at two levels (0.5 % and 1.0 % v/v), and fermentation temperature at two levels (42 and 37 °C) on CLA production, pH, bacterial cell count, antioxidant activity, and proteolysis extent were investigated.

#### 3.3.1 CLA concentration

**Fig. 1:**
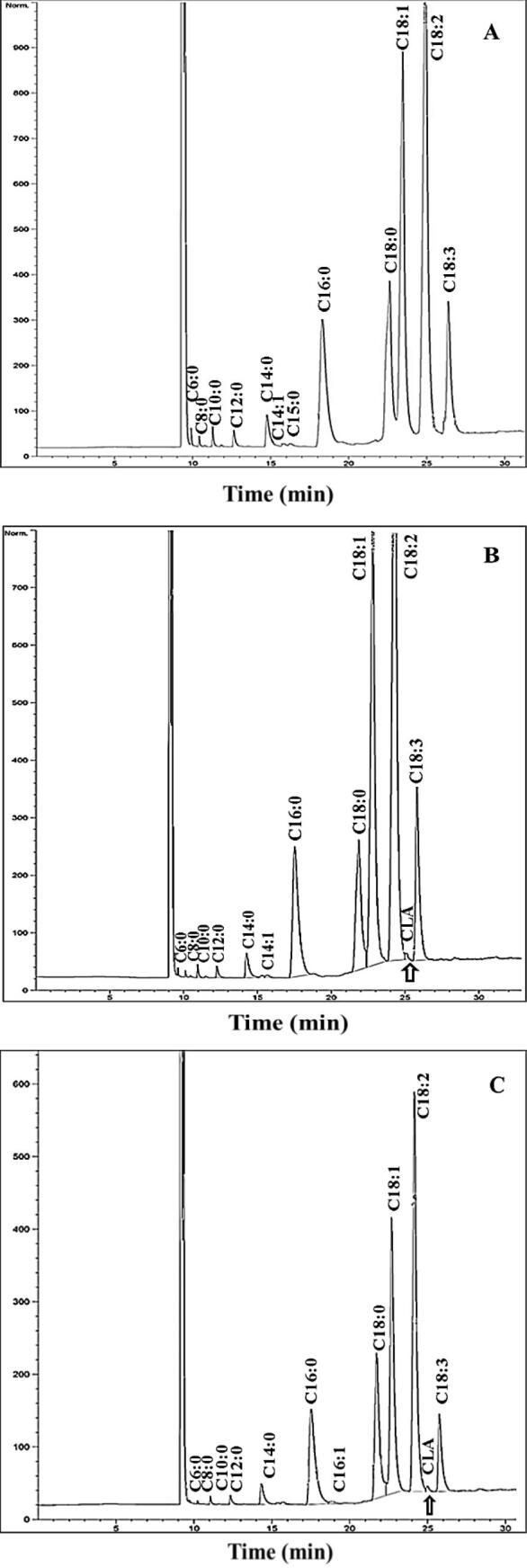
Fatty acid profiles of lipid fractions obtained from different samples in the current study; A: containing walnut oil/without fermentation, B: Fermented sample containing 0.5% lipolyzed walnut oil, and C: Fermented sample containing 1.0% lipolyzed walnut oil.

Lactic acid bacteria have a potential to lower the toxicity of free fatty acids through polyunsaturated fatty acid conversion to saturated fatty acid by formation of CLA as an intermediate metabolite (Ogawa et al., 2005). The results showed that the used starter culture was able to convert LA to CLA in the whey-based media (Fig. 1). The highest CLA content (36 mg/g fat) was observed in the sample containing 1 % lipolyzed walnut oil fermented at 42 °C for 24 h (Table 1). Indeed, the CLA production was greatly influenced by the incubation time and oil type, and the contribution of incubation time was manifestly higher than that of oil type (81.272 % *vs* 10.94 %) (Table 2).

**Table 2:** The results of analysis of variance (ANOVA) for CLA production, proteolysis, DPPH-radical scavenging activity, and cell counts in fermented whey-based beverage

	Factor	df	Contribution (%)				
			CLA	Proteolysis	DPPH	pH	Cell counts
1	Fermentation time	3	81.272	82.724	92.762	96.874	93.927
2	Oil type	1	10.94	0	0.237	0.123	0
3	Oil concentration	1	0	0	6.628	0	0
4	Temperature	1	0	13.998	0	1.037	1.366
5	Error	1	7.788	3.278	0.373	1.966	4.707
6	total	7	100 %	100 %	100 %	100 %	100 %

**Fig. 2:**
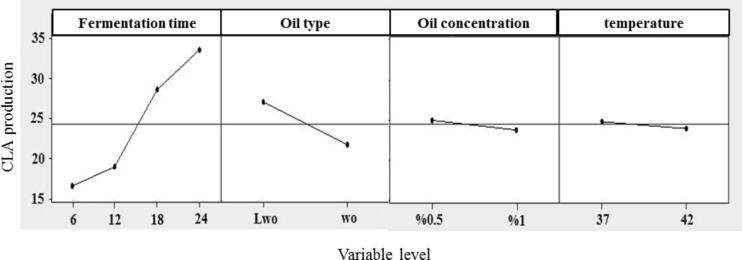
Effect of fermentation time, oil type, oil concentration, and fermentation temperature on CLA production in Taguchi method. WO: walnut oil and LWO: lipolyzed walnut oil.

The main effects of different parameters on CLA production are shown in Fig. 2. CLA production increased significantly by increasing incubation time; CLA concentration in the 24 h-fermented sample was about 2-fold higher than that in 6 h-fermented one. This is mainly due to the increased viable cell counts during fermentation (see section 3.3.3). Indeed, there was a direct and significant relationship between bacterial cell count and CLA production (r = 0.823), and the higher the number of bacterial cells, the higher was the CLA level. These results are consistent with the findings of Wang et al., 2022, Ye et al., 2013 who reported that by increasing the number of bacterial cells, the linoleate isomerase is more active to remove the toxicity factor (free linoleic acid) and subsequently convert LA to CLA. Increasing incubation time usually results in an increase in the enzyme activity and in turn CLA synthesis content. However, excessive increase in incubation time does not yield more CLA, on the other words there is not a linear correlation between incubation time and amount of produced CLA. It should be noted that CLA is toxic in large amount so there is no benefit in production of too much CLA (Kim et al., 2000). Similarly, Hosseini et al. (2015) reported that CLA production increased by increasing time to 72 h, but further incubation time (96 h) had negative effect on CLA production.

CLA production was significantly affected by the type of walnut oil. In this term, the use of lipolyzed walnut oil led to a higher CLA level in comparison to non-lipolyzed oil (Fig. 2). This could be due to the high lipolytic activity of endogenous lipases with the ability to produce free LA, and in turn LA-to-CLA conversion is more facilitated by the microorganism. Similarly, Vahvaselka & Laakso (2010) showed that by incubating oatmeal in an aqueous medium (a_w_ = 0.7) for 3 weeks, free LA was produced due to the lipolytic activity of endogenous lipase. They reported that upon the fermentation of such substrate with *P. freudenreichii *ssp. *shermanii*, LA isomerization increased significantly in the hydrolyzed oil compared to the non-lipolyzed oil. Moreover, Xu et al. (2004) revealed that hydrolyzed soy oil was a better source for production of CLA by probiotic bacteria in a milk model system.

Oil concentration had also a considerable effect on CLA production (Fig. 2). In this study, the optimum oil concentration for production of CLA was found to be 0.5 %. The suitable content of LA is different for various strains and excessive LA concentration has antibacterial effect on bacterial strains; in fact, microorganisms have different tolerance against LA (Kuhl et al., 2017). Therefore, the reduced level of produced CLA by 1 % of walnut oil can be related to the antibacterial effect of LA. However, Ando et al. (2004) reported that increasing the castor oil concentration up to 20 mg/ml caused an increase in CLA production by *L. plantarum* JCM1551. Such differences might be attributed to the differences in bacterial species and LA source/concentration.

Worthy to note that temperature did not strongly affect CLA production, and 37 °C was the optimum temperature for CLA synthesis (Fig. 2). One of the important factors affecting microorganisms’ growth and subsequently CLA synthesis is temperature. LA isomerase is a cell membrane-bound enzyme and sensitive to temperature changes (Özer & Kılıç, 2021). It has been demonstrated that the main effect of temperature is on hydrogen migration from linoleate isomerase’s double bonds, and high temperatures could destruct enzyme structure (Kim et al., 2000), while the desired temperature (37–40 °C) can induce linoleate isomerase activity and increase CLA production (Ye et al., 2013).

#### 3.3.2 Proteolysis extent

**Fig. 3:**
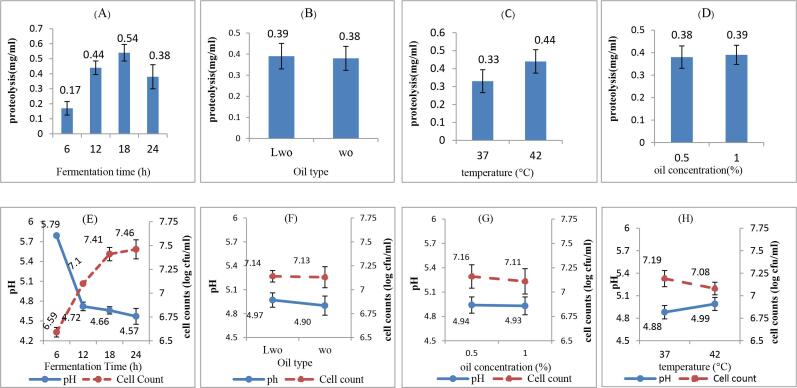
Effect of different parameters on proteolysis and pH changes during fermentation; Fermentation time (A and E), oil type (B and F), fermentation temperature (C and G), and oil concentration (D and H). WO: walnut oil, LWO: lipolyzed walnut oil.

As can be observed in Table 2, incubation time and fermentation temperature had significant effects on proteolysis and the highest contribution (82.724 %) was related to the incubation time. The proteolysis extent (as serine equivalent, mg/mL) increased remarkably from 0.17 to 0.54 mg/ml as the fermentation time rose from 6 to 18 h, likely because of microorganisms’ growth and their proteolytic activity (Fig. 3A). Indeed, proteolysis extent had a positive correlation with CLA synthesis (r = 0.823); the higher the proteolysis, the greater was the CLA production (p > 0.05). These findings are consistent with the results of Xu et al. (2005) who revealed that casein hydrolysis significantly increased CLA formation in fermented milk. However, it is necessary to point out that further incubation times (i.e., 24 h) resulted in decrease in proteolysis. This can be attributed to the consumption of free amino acids by microorganisms during growth (El-Zahar et al., 2004). Walnut oil type and concentration had no considerable effects on proteolysis (Fig. 3 B-C). In accordance to our results, Tavakoli et al. (2019) reported that fat concentration did not show a significant effect on proteolysis in probiotic yogurt. As indicated in Fig. 3D proteolysis was higher at 42 °C than 37 °C. Mazorra-Manzano et al. (2020) reported that the highest proteolysis occurred in cheese whey fermentation at 37–42 °C. Proteolytic activity during fermentation depends on different factors such as the type of bacterial strains and temperature. In this context, Pescuma et al. (2012) reported that *L. bulgaricus* and had more proteolytic activity than *S. thermophilus;* as *L. bulgaricus* activity at 42 °C is higher than that of *S. thermophilus*, so an increase in proteolysis can be expected.

#### 3.3.3 Acidity and cell count

The incubation time had the most contribution to increasing cell counts and decreasing pH (93.9 % and 96.9, respectively) (Table 2). In fact, there was an inverse relationship (r = -0.944) between cell counts and pH; generally, increase in cell count resulted in a pH decrease. As shown in Fig. 3E, rising fermentation time from 6 to 24 h led to cell counts increment and pH reduction from 6.59 to 7.46 CFU/ml and 5.79 to 4.57, respectively. This could be mainly attributed to the fact that increasing incubation time up to 24 h at 37 °C could cause a shift in microbial growth to log phase and enhancement of bacterial population, thereby leading to more acid lactic production from lactose and in turn pH reduction. Similar results have been reported by Özer and Kılıç, 2021, Xu et al., 2004 The oil type and concentration did not have considerable effects on cell counts and pH value of the system (Fig. 3F-G). On the other side, increasing temperature from 37 to 42 °C caused a decrease in cell counts (and subsequently lower CLA synthesis level) and an increase in pH value (Fig. 3H). This could be mainly ascribed to the overgrowth of *L. delbrueckii* subsp*. bulgaricus* at higher temperature, and its ability to reduce pH (and CLA content) *via* lactic acid production (Khosravi-Darani et al., 2014).

#### 3.3.4 Antioxidant activity

**Fig. 4:**
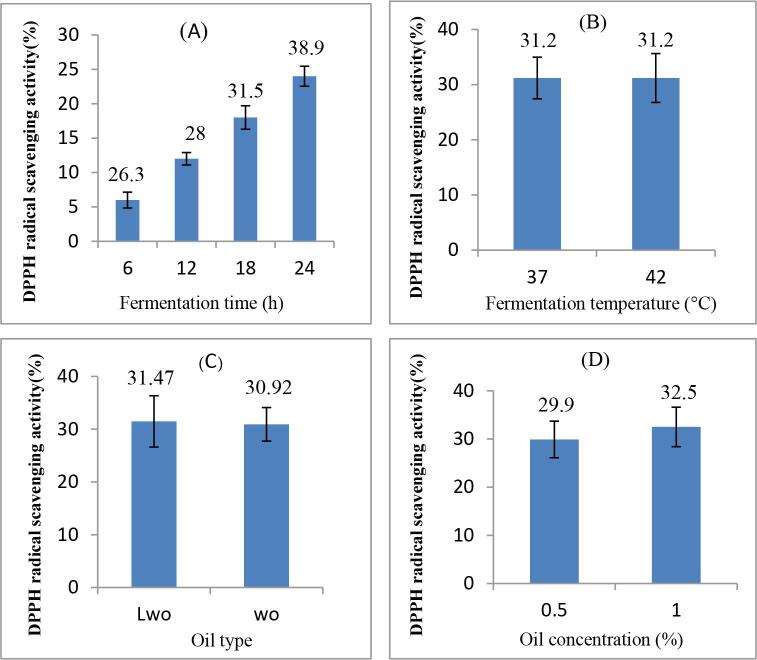
Effect of A: fermentation time, B: oil type, C: fermentation temperature and C: oil concentration on DPPH radical scavenging activity. WO: walnut oil and LWO: lipolyzed walnut oil.

Based on ANOVA results (Table 2), incubation time and oil concentration had the most effects on DPPH radical scavenging activity and the highest contribution (92.762 %) was related to the incubation time. As can be observed in Fig. 4A, by increasing incubation time from 6 to 24 h, the antioxidant activity of samples increased significantly and reached the maximum value of 38.9 %. This can be contributed to proteolysis during fermentation, leading to the liberation of antioxidant amino acids such as tyrosine, tryptophan, and phenylalanine (Yoon et al., 2019).

The walnut oil type and incubation temperature did not have considerable effects on DPPH radical scavenging activity of the sample (Fig. 4B-C). Nonetheless, Yoon et al. (2019) reported that DPPH radical scavenging activity of fermented yogurt at 22 °C was higher compared to fermented yogurt at 37 °C. Moreover, Yao et al. (2017) revealed that increasing temperature from 25 to 37 °C improved the antioxidant activity of *Eurotium cristatum*-fermented loose tea. These different results may be attributed to the differences in fermented food components, temperature range, and type of used strains.

As mentioned in Table 2, oil concentration had a remarkable effect on DPPH radical scavenging activity of samples with contribution of 6.628 %. Accordingly, the antioxidant activity of samples containing 1 % oil walnut was higher than those containing 0.5 % walnut oil (Fig. 4D), probably due the presence of tocopherol and phenolic compounds in walnut oil, with the potential to scavenge free radicals (Grajzer et al., 2020). These findings are in line with the results of Łopusiewicz et al. (2019) who reported that antioxidant activity of kefir-like fermented beverage increased significantly by increasing concentration of flaxseed oil cake.

### 3.4 Optimization of CLA synthesis during fermentation of whey-based beverage

The share of each factor in CLA production is followed the order: fermentation time > oil type > oil concentration > fermentation temperature (data in appendix). This means that the incubation time and oil type had a greater impact than other factors in CLA production. Taguchi method showed that the optimum condition for production of CLA was the use of 0.5 % lipolyzed walnut oil, fermentation time of 24 h, and fermentation temperature of 37 °C.

## 4 Conclusion

In this study, formation of CLA in fermented whey-based beverage containing walnut oil by commercial starter cultures was investigated. Although co-cultures of *L. delbrueckii* subsp*. bulgaricus* and *S. thermophilus* had suitable potential of CLA production, they were not able to synthesize CLA when applied as single cultures. This reveals the importance of synergistic effects in inserting functionality of LAB strains. In conclusion, our findings open perspectives for converting cheese whey to a value added product enriched with CLA by use of lipolyzed walnut oil as a LA source. However, the CLA concentration in the beverage manufactured in the current study may not meet the recommended daily dose of CLA for displaying significant health-promoting effects. In this term, some strain improvement programs or media enrichment may be useful for CLA overproducing. Moreover, it is necessary to confirm the storage stability of the final product containing lipolyzed oil.

## Declaration of Competing Interest

The authors declare that they have no known competing financial interests or personal relationships that could have appeared to influence the work reported in this paper.

## Data Availability

Data will be made available on request.
